# Methods and Models for Metabolic Assessment in Mice

**DOI:** 10.1155/2013/986906

**Published:** 2013-05-21

**Authors:** G. Pacini, B. Omar, B. Ahrén

**Affiliations:** ^1^Metabolic Unit, ISIB CNR, 35127 Padova, Italy; ^2^Department of Medicine, Lund University, 221 84 Lund, Sweden

## Abstract

The development of new therapies for the treatment of type 2 diabetes requires robust, reproducible and well validated *in vivo* experimental systems. Mice provide the most ideal animal model for studies of potential therapies. Unlike larger animals, mice have a short gestational period, are genetically similar, often give birth to many offspring at once and can be housed as multiple groups in a single cage. The mouse model has been extensively metabolically characterized using different tests. This report summarizes how these tests can be executed and how arising data are analyzed to confidently determine changes in insulin resistance and insulin secretion with high reproducibility. The main tests for metabolic assessment in the mouse reviewed here are the glucose clamp, the intravenous and the oral glucose tolerance tests. For all these experiments, including some commonly adopted variants, we describe: (i) their performance; (ii) their advantages and limitations; (iii) the empirical formulas and mathematical models implemented for the analysis of the data arising from the experimental procedures to obtain reliable measurements of peripheral insulin sensitivity and beta cell function. Finally, a list of previous applications of these methods and analytical techniques is provided to better comprehend their use and the evidences that these studies yielded.

## 1. Introduction

The global incidence of type 2 diabetes is predicted to grow rapidly also in the coming decades as more countries develop economically and overweight and obesity spread to populations with a genetic predisposition to the development of the disease [1]. These factors make the need for effective diabetes therapies that much greater. The development of new therapies for the treatment of type 2 diabetes requires robust, reproducible, and well validated *in vivo* experimental systems. Of particular importance are preclinical *in vivo* models, since these may select and refine experimental models for further studies and drug development in humans. There are multiple animal models of insulin resistance and decreased beta cell function including genetically deficient mice and rats as well as mouse and rat strains fed a high energy diet. Mice may provide the most ideal animal model for studies of potential type 2 diabetes therapies. Unlike larger animals, mice have a short gestational period, often give birth to many offspring at once, and can be housed in multiple groups in a single cage. This makes “in house” breeding less expensive and makes it easier to generate larger numbers to obtain well-powered studies. Gene deletion and overexpression technology have been well established in mice for over two decades, allowing researchers to create single gene mutant mice that display phenotypes which are useful in the development of type 2 diabetes therapy [2]. There are also mouse models of type 2 diabetes that have arisen due to spontaneous mutations such as the leptin deficient ob/ob and leptin receptor deficient db/db mice [3]. 

The C57/BL6 mouse strain fed a high-fat diet is a commonly used animal model in the development of potential therapies for type 2 diabetes [4]. These mice fed a high-fat diet for a short period of time (3–8 weeks) become obese and severely insulin resistant and develop postglucose load hyperglycemia, fasting hyperinsulinemia, and a diminished first phase insulin response [4, 5]. These phenotypic characteristics resemble some of the phenotypic characteristics of type 2 diabetes in humans and as such make the model a useful tool for studying potential type 2 diabetes therapies. 

The advantages of the C57/BL6 high-fat diet mouse model in studies of potential type 2 diabetes treatments are numerous. The strain itself has been inbred for hundreds of generations and its genome has been sequenced, resulting in no interindividual variation in genetic background and very little phenotypic variability. With little interindividual variation, studies can be done on fewer animals without losing statistical power which makes it a very cost effective model system. Unlike single gene mutant models of type 2 diabetes, the high-fat diet models have no deficiency of a single gene meaning that all therapeutic approaches can be applied regardless of mode of action. Additional advantages include a short generation time. Upon delivery a study can be executed in as little as five weeks and the availability of precondition high-fat diet mice, which are ready for experimentation on delivery, has also become more widely available. The high-fat diet mouse model has been extensively metabolically characterized using different metabolic tests [6]. This report aims to summarize how these tests are performed and how arising data are analyzed to confidently determine changes in peripheral insulin sensitivity and beta cell function with high reproducibility.

## 2. Experimental Tests

In general, we refer to animals weighting 20 to 25 grams. All the following considerations apply to the mouse in general, regardless of the specific strain and whether is a wild type or transgenic. Mice should be handled under anesthesia to allow serial sampling from the preorbital plexus with a lower degree of stress. Example of anesthesia is intraperitoneal injection of midazolam (0.4 mg/mouse) and a combination of fluanison (0.9 mg/mouse) and fentanyl (0.02 mg/mouse). This kind of anesthesia persists for approximately 1 h. If the experimental procedure is longer (for instance the clamp experiments; see below), the administration of anesthetics is repeated every 60 min. During the whole procedure, animals should be kept on a heating pad.

## 3. Metabolic Tests for Insulin Sensitivity and Secretion

### 3.1. Five-Hour Fasting Measurements

Sometimes, performing a dynamic test may not be possible and the investigator must only rely on a single sample drawn in a steady-state condition for which both glucose (*G*_0_) and insulin (*I*_0_) are measured. In humans, in such conditions, insulin resistance is evaluated with the HOMA model and insulin sensitivity with the log-reciprocal QUICKI formula [7] by using overnight fasting measurements. In the mouse, a long fasting means starvation with profound exhaustion of glycogen reserves and also the counterregulatory implications that this condition implies. Instead, a period of 5 hours fasting is considered to be adequate for a definition of fasting in the small rodents since it avoids massive reduction in body fat content and glycogen [8, 9].

Recent studies proposed that HOMA (and thus QUICKI) measurements refer mostly to the liver (insulin mediated inhibition of hepatic glucose production) rather than describing peripheral insulin sensitivity [10]. Extending these concepts to the mice, liver insulin sensitivity [11] can be then calculated as 1/(log *G*_0_ + log *I*_0_). With the same approach, beta cell secretion is represented by *I*_0_, while beta cell function can be described by *I*_0_/*G*_0_. Of course, these measurements refer to posthepatic hormone appearance. For a better determination of true beta cell activity, measuring C-peptide is advisable. In fact, it is equimolarly released with insulin but not degraded in the liver; thus, its peripheral concentration reflects directly the islet release of insulin.

### 3.2. Glucose Clamp

#### 3.2.1. Rationale

In humans the gold standard test is the euglycemic-hyperinsulinemic glucose clamp [12]. By maintaining glucose concentration basically constant at a target level by means of glucose infusion despite exogenously produced elevated insulin levels, this test provides an absolute index of insulin sensitivity, given by the glucose infusion rate when glucose concentration is kept at a constant steady state. The more glucose is needed, the higher is the insulin action (insulin sensitivity) on glucose uptake by peripheral tissues. If insulin levels are different among the various animals, glucose infusion must be normalized to the prevailing steady-state insulin concentration.

#### 3.2.2. Experimental Procedure

**Figure 1:**
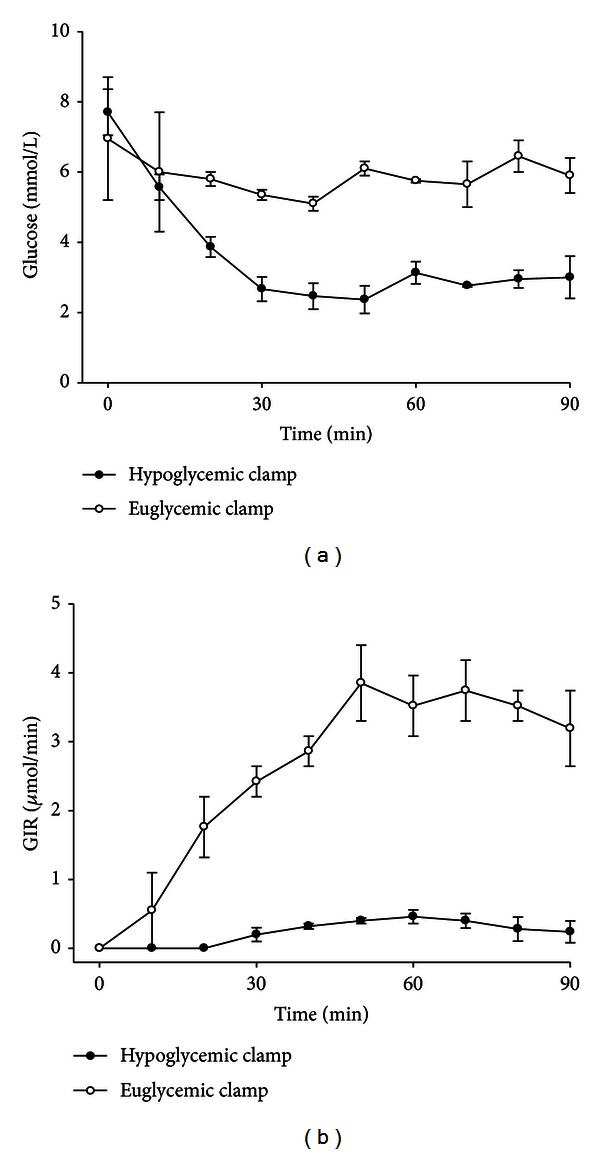
Example of glucose clamp experiments. Glucose levels and glucose infusion rates (mean ± SEM) during euglycemic clamp (aiming at the target level of 6 mmol/L glucose, *n* = 4) and hypoglycemic clamp (aiming at 2.5 mmol/L glucose, *n* = 3) in C58BL/6J mice. GIR is glucose infusion rate in the two cases.

In the anesthetized mouse, the right jugular vein and the left carotid artery are catheterized. The venous catheter is used for infusion of glucose and insulin, and the arterial catheter is used for sampling. Thirty minutes after introduction of the catheters, synthetic human insulin is infused at a rate of 60 mU/kg min^−1^ for 1 min, followed by a continuous and constant infusion of 30 mU/kg min^−1^. The volume load is 4 mL for the 1st min, followed by 2 mL/min thereafter. Blood glucose levels are determined at 5 min intervals for 120 min. A variable rate of glucose (solution of 40 g/dL) is infused to maintain blood glucose levels at 100–120 mg/dL. A blood sample is taken at 60, 90, and 120 min for determination of plasma insulin. More details can be found in previous reports [13, 14]. When the purpose of a work is examining insulin sensitivity, the glucose level during the steady state is targeting euglycemia, that is, *≈*6 mmol/L. The glucose clamp technique may, however, be used also for other purposes than determining insulin sensitivity. One such possibility is to evaluate glucose counter-regulatory mechanisms during hypoglycemia. Then, levels below baseline are targeted, for example, 2.5 mmol/L, and factors involved in the counterregulation, like glucagon, may be measured. Alternatively, the clamp may be used to estimate insulin secretory responses to standardized raised glucose levels by targeting hyperglycemia values, like 8.3 or 11.1 mmol/L. Therefore, although the main purpose of the clamp technique is usually that of maintaining euglycemia in spite of hyperinsulinemia for measurements of insulin sensitivity, the glucose clamp technique may be used for a variety of other scientific purposes with only slight modifications. Examples of eu- and hypoglycemic clamps are shown in Figure 1. The technique may also be used for distinguishing between hepatic versus peripheral insulin sensitivity by administering radiolabelled glucose in the infusate. Then, a bolus injection of [3-^3^H]glucose is given, followed by a continuous infusion of [3-^3^H]glucose throughout the study period. Blood samples are taken at steady state (60 and 90 min after the start of the infusion) for the determination of [3-^3^H]glucose concentration [15].

#### 3.2.3. Data Analysis

Insulin sensitivity is calculated as the glucose infusion rate during the second hour (*M*) divided by the mean insulin concentration at 60, 90, and 120 min (*I*), and the clamp glucose clearance per unit of insulin is calculated as *M*/*I* divided by the clamped glucose concentration. Since insulin is exogenously administered, this test does not provide any assessment of beta cell secretion for which a hyperglycemic clamp test needs to be performed. When using tritiated glucose, basal endogenous glucose production (EGP) is calculated by dividing the rate of infusion of [3-^3^H]glucose by the plasma glucose specific activity; glucose appearance at 90 min is measured by dividing the infusion rate in dpm by the plasma glucose specific activity at this time point. EGP at this time is calculated by subtracting the glucose infusion rate from the glucose appearance rate. Finally, the glucose disposal rate is calculated as the glucose appearance rate divided by the glucose concentration.

### 3.3. Intravenous Glucose Tolerance Test (IVGTT)

#### 3.3.1. Rationale

With this test, the steady state is perturbed by an injection of glucose; this directly stimulates insulin release which makes glucose to be taken up by peripheral tissues and hepatic glucose production to be inhibited. The rate of lowering of the glucose concentration for the prevailing insulin concentration is an index of insulin action. The insulin concentration is at the same time an index of the ability of the pancreas to release the hormone under the glucose stimulation.

#### 3.3.2. Experimental Procedure

**Figure 2:**
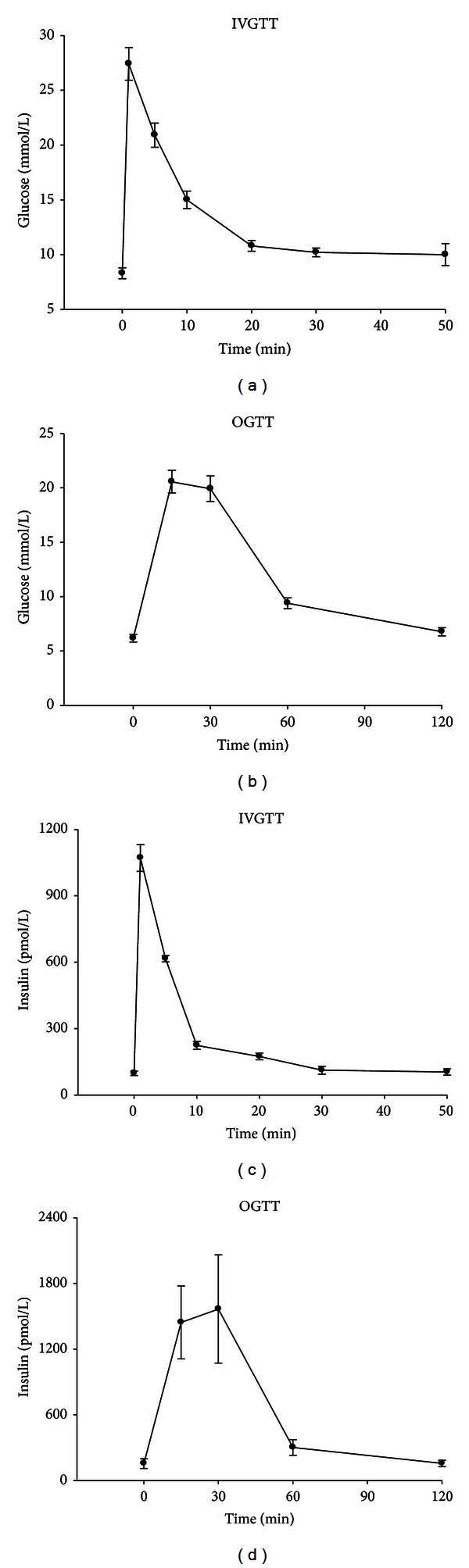
Example of outcomes from metabolic tests. Glucose and insulin levels (means ± sem) after intravenous (1 g/kg; *n* = 18) or oral (75 mg; *n* = 27) glucose administration in normal C57/BL6 mice.

In the anesthetized mouse, a blood sample is taken from the retrobulbar intraorbital capillary plexus into a 100 mL pipette that had been prerinsed in heparin solution (100 U/mL in 0.9% NaCl). Thereafter, D-glucose (solution of 10 g/dL) is injected intravenously over 3 sec at a dose of 1 g/kg in a tail vein without flushing of the 27-gauge needle after injection. The volume load is 10 mL/g body wt. The dose of 1 g/kg is quite high, because of the rapid metabolism in mice; in fact, a rise in insulin is observed only at 1 min, and rarely at 5 min, when giving lower doses of glucose such as 0.3 or 0.25 g/kg. At 1, 5, 10, 20, 30, and 50 min after injection, blood samples (75 mL each) are collected. The first sample is at 1 min, because by that time, the mixing phase of the glucose bolus could be considered terminated; the last sample is at 50 min to avoid possible influence on the measurements of awakening from anesthesia. An example is depicted in the left panel of Figure 2.

#### 3.3.3. Data Analysis

The net glucose elimination rate after the glucose injection (*K*_*G*_, the glucose tolerance index) is calculated as the slope for the interval 1–20 min after glucose injection of the logarithmic transformation of the individual plasma glucose values. Insulin sensitivity is estimated with the minimal-model technique. The model assumes first-order, nonlinear, insulin-controlled kinetics and accounts for the effect of insulin and glucose alone on net glucose disappearance. This modeling analysis that uses the whole data set from 0 to the end of the experiment provides the parameter *S*_*I*_ (insulin sensitivity index), which is defined as the ability of insulin to enhance net glucose disappearance and inhibit glucose production, and the parameter *S*_*G*_, which is the glucose effectiveness, representing net glucose disappearance per se from plasma without any change in dynamic insulin. The glucose distribution volume is calculated as the ratio of the glucose dose to the difference between the extrapolated zero intercept (a model parameter) and glucose basal level.

In order to simplify the procedure for obtaining an insulin sensitivity index, a simple formula has been developed. In previous studies, it has been shown that the tolerance index *K*_*G*_ (see above) is linearly related to the disposition index, which is a function of insulin sensitivity. In addition, it has been clearly demonstrated that the insulin sensitivity index depends upon the glucose disappearance rate and the suprabasal concentration that follows the glucose stimulation. We applied a similar approach to relate *K*_*G*_ to the metabolic parameter under the assumption that an index of insulin sensitivity should be linearly related to the ratio of *K*_*G*_/ΔAUC_ins_. ΔAUC_ins_ is defined in this case as the dynamic area under the insulin curve in the IVGTT interval 0–50 min divided by the length of the interval. This ratio, called computed insulin sensitivity (*CSI*) has been shown to be a valid surrogate of *S*_*I*_ from the minimal model for an easy use of IVGTT data. For further details on the exploitation of this method, we refer to the original publication [16], where also the additional calculation of *S*_*G*_ is clearly explained. 

Acute insulin secretion (AIR), calculated as the mean of suprabasal 1 and 5 min insulin levels, represents the early phase insulin response, while the total area under the insulin curve (AUC_insulin_) describes the total insulin release. When AUC_insulin_ is divided by AUC_glucose_, an index of glucose-mediated beta cell function can be obtained.

The IVGTT technique just described, however, requires a sufficient insulin response to the intravenous administration of glucose for a reliable estimation of the parameters. When the animals exhibit severe bluntness of glucose-stimulated insulin secretion and low serum levels of insulin in association with hyperglycemia, it is not possible to quantify *S*_*I*_ from a regular IVGTT. In fact, the suppression of insulin secretion may be so severe that hardly any suprabasal elevation of plasma insulin followed the glucose challenge is observed, making it impossible to use the model. In this case, insulin can be injected exogenously along with glucose (insulin-modified IVGTT) [17] or substances stimulating insulin release [18, 19]. However, the achieved peripheral concentration could be too high if some residual beta cell capacity has remained. In order to achieve the desired insulin levels, it is recommended to use the diazoxide-supplemented glucose-insulin test (DSGIT), for the establishment of dynamic insulin sensitivity in mice. This technique was applied for exploring insulin sensitivity in the RIP-DN HNF-1*α* mice [20]. The DSGIT is based on the potent action of diazoxide to suppress insulin secretion [17]. The procedure is the following: 30 min after anesthesia, diazoxide (25 mg/kg) is given as a subcutaneous injection. Then, 10 min later, D-glucose (1 g/kg) is given intravenously together with human insulin (doses ranging 0.1–0.4 U/kg). The volume load is in this case 10 *μ*L/g body wt. the performance of the IVGTT (sampling and assays) occurs as previously described.

It is known that insulin resistance such as in obesity is associated with an increased insulin secretion. Several studies in humans have demonstrated that a nonlinear inverse relationship (hyperbola-like) describes the physiological regulating system which allows insulin sensitivity and secretion to move in opposite directions, so that the ability of the normal subject to dispose of glucose remains relatively constant [21–23]. This constant with the IVGTT is called *disposition index* and is calculated with the product of the insulin sensitivity index times the acute insulin response, as the average of the insulin concentration during the first minutes after the glucose bolus. The hyperbolic relationship also means that a change in one of the variables is mirrored by a reciprocal change in the other variable and the understanding of this relationship is fundamental for an accurate comprehension of the nature of type 2 diabetes. Also in mice, a hyperbolic relationship was evident when plotting insulin sensitivity versus insulin secretion [17, 24] and therefore also in mice it was possible to calculate a disposition index. This has offered a tool for experimental analysis of the mechanisms regulating the interrelationships between insulin action and secretion and for studies of potential treatment modalities in mice [19]. 

The use of measurements related to circulating insulin concentration, however, does not necessarily allow information on insulin secretion, since only posthepatic insulin delivery is considered. The role of hepatic insulin extraction should in fact also be taken into account if we are interested in evaluating the beta cell function, that is, how the cell directly changes hormone release in response to changes in insulin sensitivity. To this aim, it is necessary to include in the analysis also C-peptide, which can be evaluated with either the area under the curve or concentration values at specific time points. Therefore, as a further development of the use of the hyperbolic relationship, by using *S*_*I*_ from the minimal model or *CSI* and beta cell parameters from C-peptide analysis, an index of how capable the beta cell is of adapting its secretion to changes in insulin resistance can be derived. This index thus assesses true insulin secretion in relation to insulin sensitivity; it has been called *adaptation index* and, together with the classic disposition index, provides a comprehensive picture of the mechanism of the beta cell functioning in relation to the prevailing insulin resistance. 

### 3.4. Oral Glucose Tolerance Test (OGTT)

#### 3.4.1. Rationale

This should be considered the most physiological test, since it mimics the correct route (i.e., orally) of assuming carbohydrates. The ingested glucose (usually instilled into the stomach) is absorbed in the intestinal tract and enters the splanchnic circulation and then into the systemic circulation. The increased blood glucose concentration stimulates the pancreatic beta cell to release insulin, which stimulates glucose uptake by peripheral tissues. The passage of the nutrients through the early part of the intestine stimulates the release of the gut hormones (e.g., glucose-dependent insulinotropic polypeptide, GIP, and glucagon-like peptide-1, GLP-1), which in turn augment the beta cell sensitivity to glucose, increasing the production of insulin.

#### 3.4.2. Experimental Procedure

In the 30 min period after anesthesia, a gavage tube (outer diameter 1.2 mm) is placed in the stomach to be used to administer glucose (dose 75 mg/mouse) in few seconds (standardized volume of 0.5 mL, approximate energy content 0.171 kcal). Blood samples are collected from the retrobulbar, intraorbital, capillary plexus into heparinized tubes before and either 5, 10, and 20 min or 15, 30, 60, and 90 min after oral gavage. An example is depicted in the right panel of Figure 2.

#### 3.4.3. Data Analysis

Several different possibilities exist to estimate insulin sensitivity by using empirical methods and mathematical modeling.

*ISI Comp*

This empirical method is the application in rodents of the widely used formula in humans, called also Matsuda's method [25]. It is simply calculated as 10000/(√[*G*_0_ × *I*_0_ × *G*_mean_ × *I*_mean_]), where the suffix *mean* indicates the average value of glucose and insulin concentration measurements during the whole length of the test. This formula provides a measure of insulin sensitivity.

*OGIS*

 Modeling glucose-insulin interrelationships are the basis of this method that provides a value of insulin-mediated glucose clearance that reflects insulin action. The final formula is quite complex [26], but its exploitation is easy, offering the possibility of downloading it from internet (http://webmet.pd.cnr.it/ogis/, last checked May 15, 2013). The formula requires the animal's body weight, the exact dose of administered glucose, and glucose and insulin concentration at specific samples. 

However, there is not yet validated model-derived method to assess insulin secretion or beta cell function, except the use of the area under the concentration curves (AUC). Insulin secretion can be evaluated by AUC_ins_, while beta cell function can be obtained by BCI_oral_ = AUC_ins_/AUC_gluc_. It is interesting to note that, if also GIP and/or GLP-1 have been measured during the test, it is possible to evaluate an index for the incretin effect as BCI_oral_/AUC_incr_ where AUC_incr_ is the AUC of the measured incretin concentration. If the main purpose of this study is the evaluation of the performance of the beta cell, it is again advisable to measure C-peptide, instead of insulin, under the premises that in these small animals blood withdrawn must be limited and only two compounds can be measured at one time. A more direct figure of the beta cell function can therefore be obtained from C-peptide as BCP_oral_ = AUC_cp_/AUC_gluc_. Similarly, the incretin effect on the real beta cell release is obtained by BCP_oral_/AUC_incr_. 

Even though a definition of disposition index during OGTT has never been provided, it is accepted that the product of insulin sensitivity times insulin secretion still yields a quantitative figure of the adaptive mechanism between beta cell function and insulin resistance. Albeit these indices have never been thoroughly validated during the OGTT, a possible disposition index is the product of OGIS × BCI_oral_, while the adaptation index can be derived as OGIS × BCP_oral_. 

### 3.5. Combining Oral and Intravenous Glucose Tests

An interesting and important physiological implication of oral glucose administration is the marked increase in gut hormones elicited by the glucose. These hormones are the so-called incretin hormones which stimulate insulin secretion in a glucose-dependent manner, for example, GIP and GLP-1. In humans, the incretin concept was initially demonstrated in 1964 by two studies showing that an oral glucose administration elicited a much higher insulin response than an intravenous glucose administration, in spite of a lower glucose level [27, 28]. By combining oral and intravenous glucose administration and thereby adjust the intravenous glucose infusion rate to achieve matching glucose concentration allows for a quantification of the importance of incretin factors (mainly GIP and GLP-1) for islet function after oral glucose. Such a study in mice shows that approximately 50% of the insulin secretory response (measured by C-peptide) following oral glucose administration is due to the incretin effect and not to glucose and, interestingly, that this relative contribution of incretin hormones is higher in high-fat fed mice, suggesting that the incretin factors behave as an adaptation mechanism [6].

## 4. Discussion

We have presented the most common methods to carry out metabolic studies in the mouse, particularly to obtain the main parameters related to glucose tolerance, that is, insulin sensitivity and beta cell function, and the variables describing the mechanisms of the beta cell to adapt insulin secretion to the prevailing insulin resistance. The value of the use of the tests in these animals is that the mouse is a good model for specific diseases (diabetes), for basic physiology (aging and obesity), and for the development of new drugs. All the above tests in fact can be performed in any different mouse strain and in several conceivable conditions. The overarching goal of the metabolic tests is the understanding of the etiology of diabetes and of its complications, by exploring different situations and the effects of several endogenously produced or exogenously given compounds. For instance, both the IVGTT and OGTT have been used for evaluating the insulin secretory capacity of different doses of pituitary adenylate cyclase-activating polypeptide (PACAP) [18] and glucagon-like peptide-1 (GLP-1) [19]; for the assessment of GLP-1 effects on different processes involved in glucose homeostasis [29–31]; for a metabolic picture of gastrin-releasing peptide receptor gene-deficient mice [32] and of IGF-I [33] and hepatocyte nuclear factor (HNF)-1 *α* [19] transgenic mice; for assessing the effect on insulin secretion and sensitivity of different compounds such as ghrelin [34], galanin [35], and acylation stimulating protein [36]; and for estimating the role of different nutrients on insulin resistance and beta cell function in different mouse strains [37–39]. The techniques have also been used in mice metabolically challenged with high-fat diets resulting in insulin resistance with islet adaptation [6, 24, 38]. 

The application of mouse models to the studying of metabolic derangements due to overfeeding and obesity recently assumed even more importance. In fact, with the economic, technological, and agricultural developments of the last century, access to adequate food supplies is the most widespread in human history. Unfortunately, this has, together with increasing sedentary life style with less physical activity, led to a global increase in the incidence of overweight and obesity. The global increase in overweight and obesity has created near epidemics in obesity related diseases such as cardiovascular disease and type 2 diabetes [40]. Overweight and obesity correlate strongly with diminished insulin sensitivity [41] and it is known since long time that most individuals are able to counteract the decreased insulin sensitivity by increasing insulin secretion and beta cell function [42]. In many individuals, the improvements in beta cell function are not maintained over a longer period, however, and a slow decline of beta cell function is the result [43]. The result of the decline in beta cell function is a progressive increase in fasting and postprandial hyperglycemia and the development of diabetes. Mice studies, given the relative easiness of inducing different adiposity conditions, may reveal paramount to understand the etiology, progress, and possible remedies against the obesity pandemic.

It is worth noting that no specific test has been created for the mouse, but all of them are just tests developed in humans and then adapted to animals. In some cases, like in the OGTT, the data analysis is totally similar to that for human data, while the this simplified IVGTT and the minimal model have been tailored to the size of the animal which allows only the collection of a limited number of samples. In this case, being the IVGTT in mice a sort of unexplored test, it has been necessary validating it against the gold standard (even in the animals) glucose clamp [17]. However, the IVGTT with minimal modeling assessment of insulin sensitivity and glucose effectiveness still remains a quite complex method that requires a certain skill of the operator and a wise interpretation of the modeling outputs. For this reason, a simplified, but still validated, method has been devised [16] for a reliable assessment of those fundamental parameters. It is interesting to note that, at variance with the common procedure, this simplified method developed in the mouse has been then successfully extended to humans [44].

In summary, we reviewed the main tests for metabolic assessment in the mouse and analyzed the corresponding techniques for obtaining the needed information from the data arising from the experimental procedures. We provided a list of previous applications of these methods to better comprehend their use and the evidences that these studies yielded.
